# A Lusus Naturæ

**Published:** 1888-03

**Authors:** J. N. Mendenhall


### A LUSUS NATURÆ.

*Editor Daniel's Texas Medical Journal.*—

On Feb. 21st, 1888, I delivered Mrs. C. of a well developed female child, with the exception that the left hand and most of the fore-arm were absent. It was not a case of *intra-uterine* amputation, but seemed to be an arrest of developement of the left fore-arm, that being about two inches of the *ulna*, with two rudimentary fingers on the end of it. The lady has borne four children, all sound; and there is no history of deformity in the family, except that there are several cases of rheumatism on the father's side. What caused the imperfect developement?

J. N. Mendenhall, M. D.

